# Incidence and Risk of Thyroid Dysfunction in Advanced or Metastatic Non-small Cell Lung Cancer Patients Treated with Pembrolizumab: A Meta-analysis

**DOI:** 10.7759/cureus.5997

**Published:** 2019-10-25

**Authors:** Haisam Abid, Maryam Khavandi, Nadir Siddiqui, Panadeekarn Panjawatanan, Anush Patel

**Affiliations:** 1 Internal Medicine, Bassett Medical Center, Cooperstown, USA; 2 Hematology / Oncology, Bassett Medical Center, Cooperstown, USA

**Keywords:** pembrolizumab, non-small cell lung cancer, thyroid dysfunction, incidence, risk

## Abstract

Thyroid dysfunction is one of the major side effects associated with Pembrolizumab in the treatment of advanced or metastatic non-small cell lung cancer (NSCLC). We performed a systematic review and meta-analysis of randomized clinical trials to determine its overall incidence. A literature search was conducted using the electronic database engines PubMed and Google Scholar from inception to March 2019. Eligible studies were prospective randomized clinical trials with advanced or metastatic NSCLC. The pooled incidence, risk ratio (RR), and 95% confidence interval (CI) of thyroid dysfunction were calculated using the random-effect model. Given the possibility of a between-study variance, we used the random-effect model rather than the fixed-effect model.

A total of four studies, including 1603 patients, were selected for analysis. Among patients receiving Pembrolizumab, the overall incidence of all-grade thyroid dysfunction was 19.8% (95% CI: 16.6-23.3%). Pembrolizumab was associated with a significantly increased risk of thyroid dysfunction of all grades, with a relative risk of 3.9 (95% CI: 2.08-7.42%, p= 0.084) in comparison with the controls. Therefore, there is a significant increase in developing thyroid dysfunction in advanced or metastatic NSCLC patients treated with Pembrolizumab.

## Introduction and background

Lung cancer is the leading cause of cancer-related death all over the world. The use of immunotherapy has gained precedence in the treatment of malignancies. Normally, the immune system can detect and destroy an abnormal cell using lymphocytes called T-cells. The immune system has a series of checkpoints to prevent T-cells from attacking the body's own cells. Programmed cell death 1 (PD-1) is one of these checkpoints. One hallmark of cancer is immune evasion, in which the immune system does not mount an effective antitumor response because tumor cells “hijack” the pathway to hide from T-cells [1]. PD-1 is a negative co-stimulatory receptor that is primarily expressed on the surface of activated T-cells, which blocks killing a cell when it interacts with its ligand called programmed cell death ligand (PD-L1) [2-3]. Some tumors evade the immune response by expressing these ligands on their cell surface [4]. Pembrolizumab is a monoclonal antibody directed against programmed cell death-1 receptor (anti-PD-1) and is used in the adjuvant treatment of non-small cell lung cancer (NSCLC) [5]. However, as Pembrolizumab acts to block the immune system checkpoints, it can cause T-cells to attack healthy cells, causing various autoimmune diseases referred to as immune-related adverse events (irAEs). Thyroid irAEs in patients treated with Pembrolizumab are increasingly reported as one of the most common adverse effects [6]. Given the widespread use of Pembrolizumab in advanced or metastatic NSCLC and increasing reports of thyroid dysfunction in patients treated with Pembrolizumab, we have conducted a systematic review of the literature and a meta-analysis of randomized controlled trials to evaluate the incidence and relative risk of thyroid dysfunction in patients with advanced NSCLC treated with Pembrolizumab versus controls.

## Review

Materials and methods

A literature search was conducted using the electronic database engine PubMed from inception to April 2019 for identifying randomized controlled trials in patients with advanced or metastatic NSCLC treated with Pembrolizumab. The combinations of keywords used were “non-small cell lung cancer” or “NSCLC” and “Pembrolizumab.”

Randomized controlled trials were eligible for inclusion if they reported thyroid dysfunction with the use of Pembrolizumab or controls. Articles were excluded if (1) they were not written in English or (2) no outcomes were reported. Four reviewers (HA, NS, PP, and MK) independently performed study selection according to the eligibility criteria. Disagreements were resolved by discussion with a fifth reviewer (AP).

**Figure 1:**
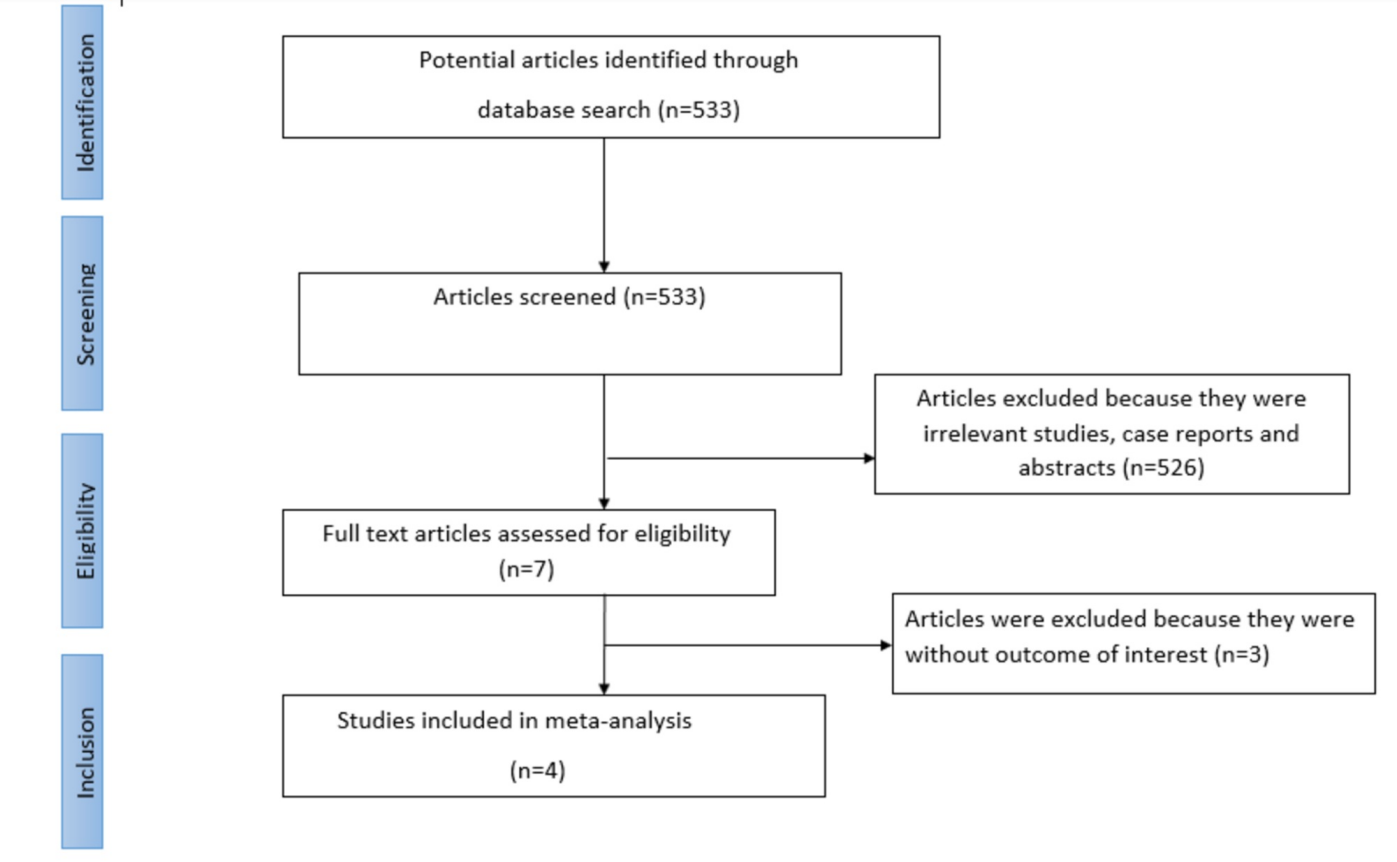
Outline of our search methodology

The following data were independently abstracted into a standardized form: study characteristics (study design, primary author, time period of study, year of publication), characteristics of the study population (total number of patients, mean age of patients, gender, tumor histology, smoking status, and previous radiotherapy and chemotherapy). A Preferred Reporting Items for Systematic Reviews and Meta-Analyses (PRISMA) flow diagram detailing the review process is shown in Figure 1.

The primary outcome analyzed in this study was thyroid dysfunction, which was defined as an abnormality of thyroid gland functioning - either hypothyroidism or hyperthyroidism. All statistical analyses were performed using Comprehensive MetaAnalysis program software 3.0 (Biostat, Englewood, NJ). Because of the possibility of a between-study variance, we used a random-effect model rather than a fixed-effect model. Forest plots were constructed to evaluate the pooled incidence and relative risk of thyroid dysfunction in advanced or metastatic NSCLC due to Pembrolizumab versus control. To determine between-study heterogeneity, we applied Cochran's Q test and *I^2 ^*statistics. A value of *I^2^* of 0%-25% represents insignificant heterogeneity, 26%-50% low heterogeneity, 51%-75% moderate heterogeneity, and 76%-100% high heterogeneity [7-8].

Results

Our search yielded a total of 562 potentially eligible articles using our search strategy. After excluding review articles, Phase I studies, single-arm Phase II studies, case reports, meta-analyses, and observational studies, we selected four randomized controlled trials (reference), including three Phase III studies and one Phase II study, which fulfilled our inclusion criteria, and their characteristics are listed in Table 1. We systematically assessed the quality of eligible studies using the published criteria [7]. The Jadad score was listed for each trial in Table 1.

**Table 1:** Characteristics of studies included in the meta-analysis

Study included	Primary author	Time period of study	Year of publication	Study design	Study quality
1.	Reck et al. Keynote-024 [9]	2014-2015	2016	Phase 3 Randomized Controlled trial	5
2.	Langer et al. Keynote-021 [10]	2014-2016	2016	Phase 2 Randomized Controlled trial	5
3.	Gandhi et al. Keynote-189 [11]	2016-2017	2018	Phase 2 Randomized Controlled trial	5
4.	Paz-Ares et al. Keynote-407 [12]	2016-2017	2018	Phase 3 Randomized Controlled trial	5

The characteristics of patients in the included studies are reported in Table 2.

**Table 2:** Characteristics of patients in included studies in the meta-analysis

Study name	Intervention	Number of patients (n)	Mean Age (years)	Male no. (%)	Female no. (%)	Squamous cell carcinoma (n)	Non-squamous cell carcinoma (n)	Smoking status Smoker Non-Smoker		Previous Radiotherapy-n (%)	Previous Chemotherapy-n (%)
Keynote-024	Pembrolizumab	154	64.5	92 (59.7)	62 (40.3)	29 (18.8)	125 (81.2)	149 (96.8)	5 (3.2)	Not reported	9 (5.8)
	Placebo	151	66	95 (62.9)	57 (27.1)	27 (17.9)	124 (82.1)	132 (87.4)	19 (12.6)	Not reported	4 (2.7)
Keynote-021	Pembrolizumab	60	62.5	22 (37%)	38 (63%)	Not reported	58 (97%)	45 (75%)	15 (25%)	Not reported	4 (7%)
	Placebo	63	63.2	26 (41%)	37 (59%)	Not reported	55 (87%)	54(86%)	9 (14%)	Not reported	5 (8%)
Keynote-189	Pembrolizumab	410	65	254 (62)	156 (38)	Not reported	394 (96.1)	362 (88.3)	48 (11.7)	28 (6.8)	30 (7.3)
	Placebo	206	63.5	109 (52.9)	97 (47.1)	Not reported	198 (96.1)	181 (87.9)	25 (12.1)	20 (9.7)	20 (11.7)
Keynote-407	Pembrolizumab	278	65	220 (79.1)	58 (20.9)	272 (97.8)	6 (2.2)	257 (92.1)	22 (7.9)	17 (6.1)	5 (1.8)
	Placebo	281	65	235 (83.6)	46 (16.4)	274 (97.5)	7 (2.5)	262 (93.2)	19 (6.8)	22 (7.8)	8 (2.8)

In total, we investigated data from 1603 patients. The mean age of patients ranged from 62 to 66 years. The majority of these patients were male (65.6%) versus female (34.4%). Reck et al. [9] and Paz-Ares et al. [12] reported patients with squamous cell carcinoma (SCC) - 66 and 546, respectively, while Gandhi et al. [11] and Langer et al. [10] did not report the total number of patients with SCC. Out of 1603 patients, 1442 (89.9%) were smokers versus non-smokers (11.1%). A total of 85 (0.05%) patients received previous chemotherapy.

**Figure 2:**
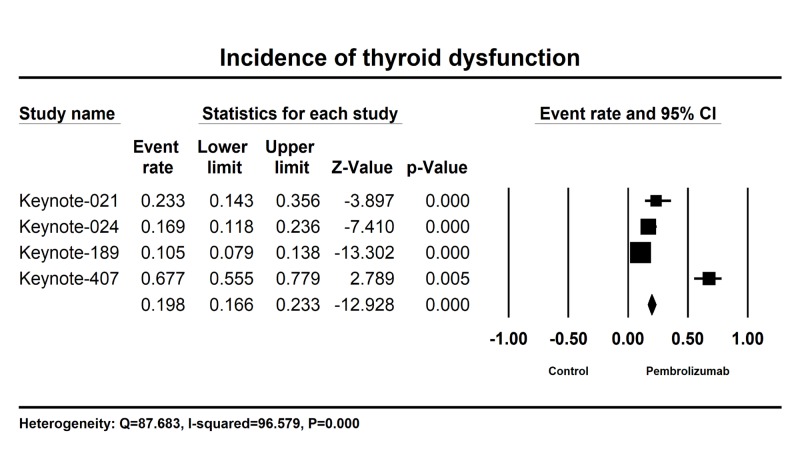
Annotated forest plot for meta-analysis of the incidence of thyroid dysfunction in advanced or metastatic non-small cell cancer patients who received Pembrolizumab. A diamond data marker represents the overall rate from each included study (square data marker) and 95% confidence interval.

A total of 1603 patients with advanced or metastatic NSCLC from four randomized controlled trials received Pembrolizumab with data of all-grade thyroid dysfunction available for analysis. The incidence among these trials showed that all-grade thyroid dysfunction ranged from 10.5% to 67.7%, with the lowest incidence (10.5%) observed in Keynote-189 and the highest incidence (67.7%) in Keynote-407. The pooled incidence of all-grade thyroid dysfunction was 19.8% (95% CI: 16.6-23.3%) among patients receiving Pembrolizumab from all these trials (Figure 2). Meta-analysis showed that heterogeneity (Q = 87.683, *I^2^* = 96.579, P=0.000) exists among these trials.

**Figure 3:**
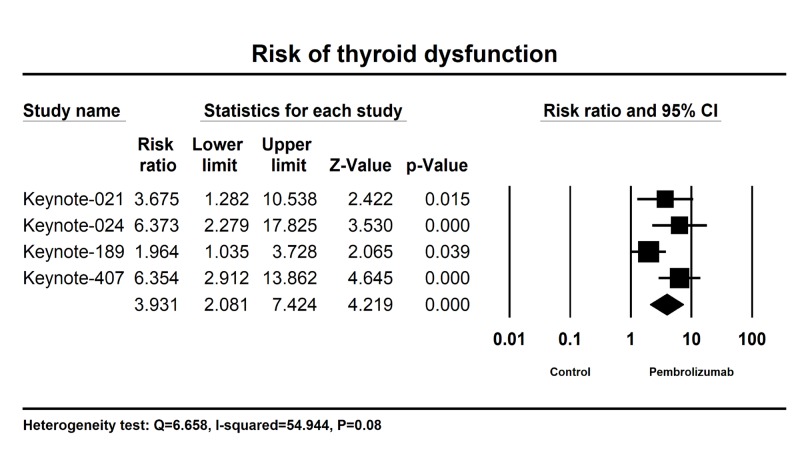
Relative risk of thyroid dysfunction associated with Pembrolizumab versus control. The summary relative risks (RR) of thyroid dysfunction were calculated using the random-effects model. RR and 95% confidence intervals for each study and the final combined result are displayed numerically on the left and graphically as a forest plot on the right.

We also calculated the relative risk (RR) of thyroid dysfunction in patients with advanced or metastatic NSCLC receiving Pembrolizumab versus control. Results showed RR was 3.93 (95% CI: 2.08-7.42, P < 0.001) for all-grade thyroid dysfunction (Figure 3). Thus, Pembrolizumab is associated with a significantly increased risk of thyroid dysfunction in patients with advanced or metastatic NSCLC.

Discussion

In this systematic review, we have determined the overall risk of thyroid dysfunction associated with the use of Pembrolizumab with advanced or metastatic NSCLC cancer. We have demonstrated a high incidence of thyroid dysfunction 19.8% (95% CI: 16.6-23.3% associated with Pembrolizumab. In addition, the risk of thyroid dysfunction in comparison with controls is 3.93 (95% CI: 2.08-7.42, P < 0.001). Adequate monitoring and aggressive management of thyroid dysfunction is essential for many of these patients, as it can be life-threatening. With the widespread use of Pembrolizumab in NSCLC patients, early recognition and effective management become more important.

Hypophysitis and thyroid disorders are the most common endocrinopathies associated with immune checkpoint inhibitors [13-15]. Hypophysitis is more commonly associated with cytotoxic T-lymphocyte-associated protein 4 (CTLA-4) inhibitors, such as ipilimumab, whereas thyroid disorders are more common in patients receiving antibodies against programmed cell death-1 receptor (anti-PD-1) such as Pembrolizumab and Nivolumab [16].

Hypothyroidism and thyrotoxicosis due to the inappropriate activation of T-cells leading to the destruction of the thyroid gland are the most frequent clinical presentations. The risk of developing thyroid dysfunction is high within the first few weeks after starting Pembrolizumab. The time frame for the development of thyrotoxicosis progressing to hypothyroidism is usually brief, often following the first Pembrolizumab dosing. High-grade thyroid dysfunction is very rare with Pembrolizumab [17].

Pembrolizumab-induced thyroid dysfunction can present in many different ways. Usually, it presents as hypothyroidism due to the autoimmune destruction of the thyroid gland. Sometimes, it can also manifest as hyperthyroidism or transient hyperthyroidism followed by longstanding hypothyroidism. Most commonly, the clinical presentation is non-specific such as fatigue or generalized weakness, which is why clinicians should have a high degree of suspicion for an accurate diagnosis. There is no antibody to diagnose Pembrolizumab-associated thyroid dysfunction. Since the clinical presentation is similar, we should distinguish primary hypothyroidism from secondary hypothyroidism due to hypophysitis because of the difference in their management. It has been recommended to obtain baseline thyroid-stimulating hormone (TSH) level prior to initiating Pembrolizumab [18].

The treatment of thyroid dysfunction depends upon the severity and presence or absence of symptoms [19]. In patients with mild disease and absence of symptoms, no treatment is indicated, and they can safely continue Pembrolizumab but close monitoring of thyroid function tests (TFTs) is recommended. For patients with mild symptoms of hypothyroidism or in asymptomatic patients with TSH levels that persist > 10 mIU/L (measured four weeks apart), the treatment of choice is levothyroxine and the close monitoring of TFTs, and the recommendation is to hold Pembrolizumab until symptoms resolve. While for mild hyperthyroidism, the treatment of choice is a beta-blocker and to hold Pembrolizumab until symptoms return to baseline. For severe cases, such as a thyroid storm, hospitalization, intravenous (IV) steroids (1-2 mg/kg/day), and endocrinologist consultation are recommended [20]. According to Brahmer et al., Pembrolizumab can be resumed after the patient is stabilized with appropriate therapy even in severe cases [19].

The following limitations of our systematic review are worth mentioning. Despite performing a comprehensive literature search, we cannot exclude the possibility of having missed a relevant article. There are statistical heterogeneities in our meta-analysis, which may have affected the incidence of thyroid dysfunction in this population.

## Conclusions

Pembrolizumab is associated with a significantly increased risk of thyroid dysfunction in patients with advanced or metastatic NSCLC as compared to controls.
